# Comparison of radiofrequency ablation and antiarrhythmic drug for the treatment of atrial fibrillation: A protocol for systematic review and meta-analysis

**DOI:** 10.1097/MD.0000000000032184

**Published:** 2022-12-23

**Authors:** Junping Deng, Yujun Gan, Yuxuan Shan, Hui Guo

**Affiliations:** a Department of Cardiology, Linfen People’s Hospital, Linfen, Shanxi Province, China.

**Keywords:** antiarrhythmic drug, atrial fibrillation, meta-analysis, radiofrequency ablation, review

## Abstract

Antiarrhythmic drugs and radiofrequency ablation are first-line treatments of atrial fibrillation, however, there exists a paucity of data regarding the potential benefit of different catheter ablation technologies versus antiarrhythmic drugs as an early rhythm strategy. We performed a protocol for systematic review and meta-analysis to compare the efficacy and safety of radiofrequency ablation and antiarrhythmic drugs for the treatment of atrial fibrillation.

**Methods:**

This review protocol is registered in the International Prospective Register of Systematic Reviews (PROSPERO: CRD42022375095). Additionally, this review will adhere to the Preferred Reporting Items for Systematic Reviews and Meta-Analyses Protocols 2015 Statement. A computerized literature search will be performed in the following electronic databases from their inceptions to November 2022: PubMed, EMBASE, MEDLINE, Cochrane Central Register of Controlled Clinical Trials, China Knowledge Resource Integrated Database, Wanfang Data Information, and Weipu Database for Chinese Technical Periodicals. The risk of bias will be assessed independently by 2 authors using the Cochrane tool of risk of bias. All statistical analyses will be conducted using the software program Review Manager version 5.3.

**Results:**

The results of this systematic review will be published in a peer-reviewed journal.

**Conclusion:**

This study provides evidence of the comparison of radiofrequency ablation and antiarrhythmic drugs for the treatment of atrial fibrillation.

## 1. Introduction

Atrial fibrillation (AF) is the most common type of cardiac arrhythmia.^[1,2]^ With the ever-aging population, the prevalence of AF is also increasing. In AF, the upper chambers of the heart do not function correctly as a result of abnormal electrical signaling. It can be characterized by rapid and irregular atrial depolarizations with a discrete lack of P waves on electrocardiograms.^[3,4]^ As a result, the blood in the atria remains static and can promote blood clot formation and increase the risk of stroke.^[5,6]^ This can cause detrimental symptoms, impair functional status and reduce the quality of life. In recent times, advancements in medical technology have helped us gain a greater understanding of AF and the mechanisms of its onset. As a result, many novel pharmacological and nonpharmacological therapies have been developed that can control or potentially prevent AF.^[7,8]^

For the majority of patients that do not require immediate cardioversion, anti-arrhythmic drug therapies can potentially be utilized. Antiarrhythmic drugs have been highly effective in converting AF to normal sinus rhythm if promptly administered following onset of AF and at an adequately high dosage.^[9,10]^ However, other methods of interventions are required if the patient is presented with other conditions such as unstable angina, acute myocardial infarction or any other abnormal ventricular response that are related to preexcitation syndrome.

A beneficial effect of radiofrequency ablation (RFA) on quality of life has been suggested by several studies,^[11,12]^ but it is unclear whether the effect of RFA on quality of life is sustained, because long-term follow-up data have been limited. The optimal therapeutic approach for AF is still debated and no randomized controlled trials (RCTs) have been sufficiently powered to address these issues. We performed a protocol for systematic review and meta-analysis to compare the efficacy and safety of RFA and antiarrhythmic drug for the treatment of AF.

## 2. Methods

This review protocol is registered in the International Prospective Register of Systematic Reviews (PROSPERO: CRD42022375095). Additionally, this review will adhere to the Preferred Reporting Items for Systematic Reviews and Meta-Analyses Protocols 2015 Statement.^[13]^ No ethical statement will be required for the performance of this review as this study is based on published trials.

### 2.1. Search strategy

A computerized literature search will be performed in the following electronic databases from their inceptions to November 2022: PubMed, EMBASE, MEDLINE, Cochrane Central Register of Controlled Clinical Trials, China Knowledge Resource Integrated Database, Wanfang Data Information, and Weipu Database for Chinese Technical Periodicals. A combination of subject words and free text words will be applied in the searches. The language is limited to Chinese and English. The literature search strategy is summarized for PubMed in Table 1.

**Table 1:** Search strategy in PubMed

#1 atrial fibrillation [Title/Abstract]
#2 auricular fibrillation [Title/Abstract]
#3 atrial tachyarrhythmia [Title/Abstract]
#4 #1 OR #2 OR #3
#5 antiarrhythmic drug [Title/Abstract]
#6 propafenone [Title/Abstract]
#7 flucaramine [Title/Abstract]
#8 dophilite [Title/Abstract]
#9 lbutilide [Title/Abstract]
#10 amiodarone [Title/Abstract]
#11 cedilanid [Title/Abstract]
#12 #5 OR #6 OR #7 OR #8 OR #9 OR #10 OR #11
#13 radiofrequency ablation [Title/Abstract]
#14 interventional therapy [Title/Abstract]
#15 catheter ablation [Title/Abstract]
#16 electrofulguration [Title/Abstract]
#17 #13 OR #14 OR #15 OR #16
#18 #4 AND #12 AND #17

### 2.2. Selection of eligible studies

**Figure 1.**
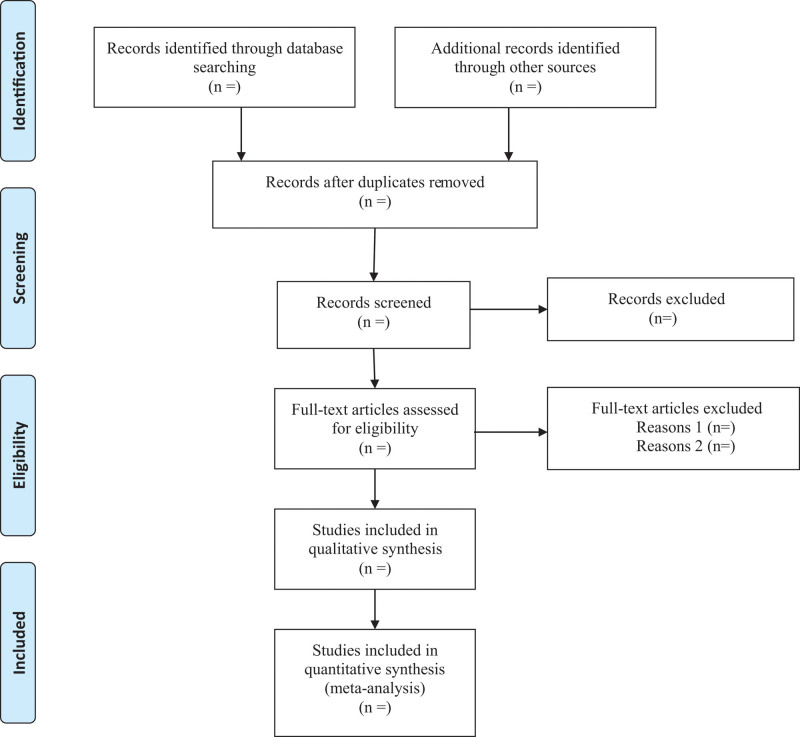
PRISMA flow diagram for study selection.

Two reviewers will independently screen the titles and abstracts of the retrieved articles. We will also acquire the full text for screening to evaluate the eligibility for inclusion when necessary. Any disagreements will be resolved by discussion among reviewers. The process and results of the studies selection will be presented in a flow chart with Fig. 1. The inclusion/exclusion criteria are as following: types of studies: RCTs will be included in this systematic review regardless of publication status and language. Quasi-RCTs and nonrandomized studies will be excluded; types of participants: participants with AF, who are 18 years or older, are included regardless of their age, sex, or race; types of interventions: intervention group receive RFA and control group receive drug therapy. There is no limitation on the device, dosage, or duration of treatment; types of outcome measures: the main outcome measures are recurrence of AF and quality of life; the additional outcomes are adverse events such as stroke, bleeding and all-cause mortality.

### 2.3. Data extraction

For all studies included, 2 review authors will independently extract the relevant information using a standard data extraction table. Information will include publication of year, author, participants, intervention, control, duration of intervention, outcomes, and methodologic characteristics. Disagreements will be resolved by discussion by arbiter.

### 2.4. Risk of bias assessment

The risk of bias will be assessed independently by 2 authors using the Cochrane tool of risk of bias (V.5.1.0).^[14]^ The following items will be assessed: random sequence generation (selection bias), allocation concealment (selection bias), blinding (performance bias and detection bias), incomplete outcome data (attrition bias), selective outcome reporting (reporting bias), and other bias. The judgments of evaluated domains will include high, low, and unclear. Disagreements will be resolved by discussion by arbiter.

### 2.5. Statistical analysis

Differences between the intervention and control groups will be assessed. Mean differences (MDs) with 95% confidence intervals (CIs) will be used to measure the effects of treatment for continuous data. We will convert other forms of data into MDs. For outcome variables on different scales, we will use standard MDs with 95% CIs. For dichotomous data, we will present the treatment effects as relative risks with 95% CIs, and other binary data will be converted into relative risk values. All statistical analyses will be conducted using the software program Review Manager version 5.3 (Copenhagen, The Nordic Cochrane Centre, the Cochrane Collaboration, 2014) for Windows. We will contact the corresponding authors of the studies with missing information to acquire and verify the data, whenever possible. When appropriate, we will pool the data across studies to conduct a meta-analysis using fixed or random effects. Subgroup analyses will be performed to identify the possible causes in cases of heterogeneity (defined by results of tests of heterogeneity that indicate *P* < .1 via Chi-squared tests and Higgins *I*^2^ ≥ 50%).

Sensitivity analysis will be also applied to evaluate the robustness and reliability of the combined results of included studies. Methodological quality, heterogeneity, studies quality, and sample characteristic will be considered. We will conduct analysis of Egger publication bias plot and Begg funnel plot with pseudo 95% confidence limits to determine the publication bias in all the literature with sufficient studies (more than 10 trials).

### 2.6. Grading quality of evidence

Furthermore, for grading the strength of the evidence for all outcomes from the included data, the Grading of Recommendations Assessment, Development, and Evaluation method or an equivalent methodology will be clearly described and documented by 2 independent researchers.

## 3. Discussion

AF is the main cause of stroke, and is associated with higher rates of mortality and cardiovascular disease.^[15,16]^ The major causes of death in patients with AF are progressive heart failure, cardiac arrest, and stroke.^[17,18]^ In addition to the risk factors and lifestyle management (eg, alcohol, obesity, sleep apnea, lack of exercise) as well as stroke prevention,^[19]^ rate control and rhythm control are the most common strategies employed for treating AF.^[3,20]^ Antiarrhythmic drugs and RFA are first line treatments of AF, however, there exists a paucity of data regarding the potential benefit of different catheter ablation technologies versus antiarrhythmic drugs as an early rhythm strategy. To the best of our knowledge, this is the first meta-analysis to compare the efficacy and safety of RFA and antiarrhythmic drugs for the treatment of AF. However, due to the limitations of the present review, more high-quality, multicenter RCTs are needed to further confirm the conclusion.

## Author contributions

**Conceptualization:** Yujun Gan.

**Formal analysis:** Yuxuan Shan.

**Methodology:** Hui Guo.

**Writing—original draft:** Junping Deng.
